# TNF-Alpha rs1800629 Polymorphism Is Not Associated with HPV Infection or Cervical Cancer in the Chinese Population

**DOI:** 10.1371/journal.pone.0045246

**Published:** 2012-09-13

**Authors:** Ning Wang, Duo Yin, Shulan Zhang, Heng Wei, Shizhuo Wang, Yang Zhang, Yanming Lu, Shuyan Dai, Wei Li, Qiao Zhang, Yao Zhang

**Affiliations:** 1 Department of Gynecology and Obstetrics, Shengjing Hospital of China Medical University, Shenyang, China; 2 Department of Breast Surgery, Shengjing Hospital of China Medical University, Shenyang, China; University of Leicester, United Kingdom

## Abstract

**Background:**

While HPV infection is the main cause of cervical cancer, genetic susceptibility to HPV infection is not well understood. Tumor necrosis factor alpha (TNF-alpha), involved in the defense against HPV infection, plays an important role in cervical cancer progression and regression. The aim of this study was to investigate the relationship between the TNF-alpha rs1800629 polymorphism and risk of HPV infection or cervical cancer.

**Methods:**

Three groups were involved in this study of Chinese women. Group 1 consisted of 285 high risk HPV positive cervical cancer patients, Group 2, 225 high risk HPV positive patients without cervical cancer, and Group 3, 318 HPV negative women with no cervical cancer. Blood samples were obtained from all patients and genotyped by PCR-RLFP. Fifty randomly selected samples were further sequenced.

**Results:**

The allele and genotype distributions of the TNF-alpha rs1800629 polymorphism were not significantly different between each of the groups (P>0.05). There are no significant relationship between rs1800629 polymorphism and high risk HPV infection (OR  = 0.649, 95% CI: 0.253–1.670, P = 0.371), cervical cancer (OR  = 0.993, 95% CI: 0.376–2.618, P = 0.988), or cervical cancer with HPV infection (OR  = 0.663, 95% CI: 0.250–1.758, P = 0.409).

**Conclusions:**

We demonstrated that there is no association between TNF rs1800629 polymorphism and the HPV infection, or cervical cancer with HPV infection.

## Introduction

Cervical cancer remains the second most common cancer among women worldwide [1]. Infection by an oncogenic human papillomavirus (HPV) is a risk factor for developing cervical cancer [2]. Immune responses to HPV infection within the cervical epithelium play an important role in the pathogenesis of cervical cancer.

Several cytokines that modulate the immunologic response have been implicated in the development of cancer [3]. Tumor necrosis factor-alpha (TNF-α), secreted mainly by activated macrophages, is an extraordinarily pleiotropic cytokine with a central role in immune homeostasis, inflammation, and host defense [4], [5]. TNF-α is involved in the defense against HPV infection, modulating viral replication [6].

The role of TNF-α in cancer is not well understood. Deregulated TNF expression within the tumor microenvironment appears to favor malignant cell tissue invasion, migration, and ultimately metastasis formation [7]. There is also evidence, however, that TNF-α may promote the development and spread of the cancer [8], [9]. The role of TNF-α in tumor promotion is supported by the TNF-α-deficient mouse model, in which TNF-α^−/−^ mice are resistant to the development of benign and malignant skin tumors. TNF modulation may contribute to regulation of cell inflammation, and the subsequent development of malignant disease [10]. Since the malignant development of cervical cancer is induced by persistent viral infection, we focus on the TNF gene, which may be involved in the susceptibility to HPV infection and development of cervical cancer.

**Table 1:** Baseline clinical characteristics of involved groups.

	Group 1 (n = 285)	Group 2 (n = 225)	Group 3 (n = 318)	
	No (%)	No (%)	No (%)	
Age				
≤35	57 (20.0)	53 (23.6)	78 (24.5)	0.224
36–50	161 (56.5)	136 (60.4)	174 (54.7)	
>50	67 (23.5)	36 (16)	66 (20.8)	
Smoking				
Never	270 (94.7)	208 (92.4)	307 (96.5)	0.108
ever	15 (5.3)	17 (7.6)	11 (3.5)	
HPV status				
HPV+	285 (100)	225 (100)	0 (0)	
HPV−	0 (0)	0 (0)	318 (100)	
Clinical stage^*^				
Stage 0	142 (49.8)			
Stage I	108 (37.9)			
Stage II	28 (9.8)			
Stage III	6 (2.1)			
Stage IV	1 (0.4)			

Note: ^*^ According to the International Federation of Gynecology and Obstetrics classification.

TNF-α is regulated at the transcriptional level [11] and the rs1800629 polymorphisms within the TNF-α promoter region have been associated with the level of TNF-α [12]. The association of rs1800629 polymorphism and cervical cancer has been widely studied, but the results are contradictory [13], [14], [15], [16]. The current study was conducted to investigate the distribution of rs1800629 polymorphism and its relationship with HPV infection and cervical cancer.

**Table 2:** Allele and Genotype distribution in different groups.

	Group 1 (n = 570)		Group 2 (n = 450)		Group 3 (n = 636)	
	No (%)	P _G1–G2_	No (%)	P _G2–G3_	No (%)	P _G1–G3_
Allele						
G	524 (91.9)	0.271	404 (89.8)	0.287	583 (91.7)	0.916
A	46 (8.1)		46 (10.2)		53 (8.3)	
Genotype	(n = 285)		(n = 225)		(n = 318)	
GG	247 (86.7)	0.551	188 (83.6)	0.627	274 (86.2)	0.980
GA	30 (10.5)		28 (12.4)		35 (11.0)	
AA	8 (2.8)		9 (4.0)		9 (2.8)	

Note: G1–G2: comparison between Group 1 and Group 2; G2–G3: comparison between Group 2 and Group 3; G1–G3: comparison between Group 1 and Group 3; p value for test of HWE was 0.0013.

**Table 3:** ORs between Group 2 (HPV negative without cervical cancer) and Group 3 (HPV negative without cervical cancer).

Genotype	Group 2 (n = 225)	Group 3 (n = 318)	OR^*^ (95% CI)	P	OR^*^ (95% CI)	P
	No. (%)	No. (%)				
GG	188 (83.6)	274 (86.2)	Reference		1.540(0.599–3.960)	0.371
GA	28 (12.4)	35 (11.0)	0.880(0.516–1.502)	0.640	1.356(0.473–3.889)	0.571
AA	9 (4.0)	9 (2.8)	0.649(0.253–1.670)	0.371	reference	
GG+GA	216 (96.0)	309 (97.2)	Reference		1.517 (0.591–3.893)	0.386
AA	9 (4.0)	9 (2.8)	0.659(0.257–1.692)	0.386	reference	
GA+AA	37 (16.4)	44 (13.8)	Reference		0.822 (0.510–1.325)	0.421
GG	188 (83.6)	274 (86.2)	1.216(0.755–1.961)	0.421	reference	

Note: * Data were calculated by multivariate regression analysis, adjusting for age and smoking status.

**Table 4:** ORs comparison between Group 1 (cervical cancer) and Group 3 (HPV negative with no cervical cancer).

Genotype	Group 1 (n = 285)	Group 3 (n = 318)	OR^*^ (95% CI)	P	OR^*^ (95% CI)	P
	No. (%)	No. (%)				
GG	247 (86.7)	274 (86.2)	reference		1.007(0.382–2.657)	0.988
GA	30 (10.5)	35 (11.0)	0.932(0.554–1.567)	0.790	0.791(0.321–2.747)	0.908
AA	8 (2.8)	9 (2.8)	0.993(0.376–2.618)	0.988	reference	
GG+GA	277 (97.2)	309 (97.2)	reference		1.000 (0.380–2.632)	1.000
AA	8 (2.8)	9 (2.8)	1.000(0.380–2.634)	1.000	reference	
GA+AA	38 (13.3)	44 (13.8)	reference		0.944 (0.591–1.509)	0.811
GG	247 (86.7)	274 (86.2)	1.059(0.663–1.692)	0.811	reference	

Note: ^*^Data were calculated by multivariate regression analysis, adjusting for age and smoking status.

**Table 5:** ORs comparison between Group 1 (cervical cancer) and Group 2 (HPV positive without cervical cancer).

Genotype	Group 1 (n = 285)	Group 2 (n = 225)	OR^*^ (95% CI)	P	OR^*^ (95% CI)	P
	No. (%)	No. (%)				
GG	247 (86.7)	188 (83.6)	Reference		1.508(0.569–3.998)	0.409
GA	30 (10.5)	28 (12.4)	0.785(0.451–1.368)	0.393	1.184(0.398–3.523)	0.761
AA	8 (2.8)	9 (4.0)	0.663(0.250–1.758)	0.409	reference	
GG+GA	277 (97.2)	216 (96.0)	Reference		1.468 (0.555–3.882)	0.439
AA	8 (2.8)	9 (4.0)	0.681(0.258–1.802)	0.439	reference	
GA+AA	38 (13.3)	37 (16.4)	Reference		0.755 (0.460–1.239)	0.266
GG	247 (86.7)	188 (83.6)	1.324 (0.807–2.173)	0.266	reference	

Note: ^*^Data were calculated by multivariate regression analysis, adjusting for age, smoking status and HPV status.

## Materials and Methods

### Study subjects

From 2008 to 2010, all patients treated at the Department of Obstetrics and Gynecology, Shengjing Hospital of China Medical University were evaluated for entry into this study. Patients were divided into 3 groups. Group 1 consisted of 285 patients diagnosed with cervical cancer and high risk HPV infection (including HPV 16, 18, 31, 33, 35, 39, 45, 51, 52, 56, 58, 59 and 68). Group 2 consisted of 225 patients diagnosed with high risk HPV infection, but without any abnormal cervical cytological or pathological change. Group 3 was composed of 318 healthy Chinese women presenting for routine healthy screening at our Health Check Center. Group 3 patients had no history of HPV infection, cervical neoplastic disease, or evidence of cervical pathology. Patients in Group 3 had a normal cervical cytology on at least two consecutive annual examinations. Women with any history of malignant disease were excluded from Group 3. All patients were Chinese. Informed written consent and 5 ml of peripheral blood were obtained from each participant. The study protocol was approved by the Ethics Committee of Shengjing hospital. HPV status was detected as previously described [17].

### Genotyping of the rs1800629 polymorphism

Genomic DNA was extracted from peripheral blood leukocytes (Tiangen Blood Genome Kit). Genotyping was determined by polymerase chain reaction-restriction fragment length polymorphism (PCR-RFLP) analysis, which was based on the method of Cabrera et al. [18]. The PCR products from 50 random patient samples were further sequenced to confirm the PCR-RFLP genotyping results.

### Statistical analysis

The association between *TNF*-α rs1800629 polymorphism and HPV infection and cervical cancer was estimated using odds ratio analysis (ORs) and their 95% CIs calculated by multivariate logistic regression. All the ORs were adjusted for age and smoking status. Hardy-Weinberg equilibrium analysis was performed with the Chi-square test. The Chi-square test was used to compare variables. A p-value of 0.05 was used as the criterion of statistical significance. All analyses were conducted using SPSS software (version 13.0; SPSS, Inc., Chicago, IL).

## Results

The basic clinical characteristics of the three groups are summarized in Table 1. The distribution of age and smoking was not significantly different between the 3 groups (P = 0.224 and 0.108, respectively). Patients in the cervical cancer group (Group 1) were all HPV positive. Cervical cancer was staged according to the International Federation of Gynecology and Obstetrics classification and 49.8, 37.9, 9.8 and 2.1% of patients had stage 0, I, II and III diseases, respectively. Only 1 patient had stage IV cervical cancer. As defined, patients in Group 2 were all HPV positive and had no abnormal cervical findings.

The PCR-RFLP analysis results of 50 random samples were all confirmed by sequencing (Fig. S1). The genotype frequencies in all groups did not conform to the Hardy-Weinberg equilibrium (P = 0.0013). The allelic frequencies of the A allele in Groups 1, 2 and 3 were 8.1, 10.2 and 8.3%, respectively. No significant differences of allelic frequencies were found between groups (P_G1–G2_ = 0.271, P_G2–G3_ = 0.287 and P_G1–G3_ = 0.916, Table 2). No significant difference of genotype distribution was observed between the groups (P_G1–G2_ = 0.551, P_G2–G3_ = 0.627 and P_G1–G3_ = 0.980, Table 2). There was no difference in the distribution of genotypes among patients with different clinical stages of cervical cancer (Group 1) (P = 0.575).

In further multivariate regression analysis, no significant association was observed in the comparison of genotypes after adjusting for age, smoking, and HPV infection status. Comparing Groups 2 and 3, we found no significant relationship between TNF rs1800629 polymorphism and HPV infection (AA vs. GG: OR  = 0.649, 95% CI: 0.253–1.670, P = 0.371. Table 3). Comparing Groups 1 and 3, we found there was no significant relationship between TNF rs1800629 polymorphism and cervical cancer (AA vs. GG: OR  = 0.993, 95% CI: 0.376–2.618, P = 0.988. Table 4). Comparing Groups 1 and 2, we found no significant association between TNF rs1800629 polymorphism and cervical cancer with HPV infection (AA vs. GG: OR  = 0.663, 95% CI: 0.250–1.758, P = 0.409. Table 5). No associations were found in combined genotypes (AA vs. GG+GA or GG vs. GA+AA) with cervical cancer risk (Table 3–5).

## Discussion

In this study, we evaluated patients with high risk HPV and cervical cancer (Group 1), with high risk HPV and no cervical cancer (Group 2), and with neither high risk HPV or cervical cancer (Group 3) We evaluated the relationship between rs1800629 polymorphisms and HPV infection and cervical cancer. We did not observe a significant relationship between rs1800629 polymorphisms and cervical cancer or HPV infection.

Although several studies have indicated that polymorphisms in the promoter region of rs1800629 may be a contributing factor to the development of cervical cancer [14], [15], [19], [20], [21], the results have been contradictory. It is not clear why studies upon identical malignancies produced divergent results. Differences between study design, investigated populations, and analytic method used may have contributed to these inconsistencies. Large prospective studies with matched case-control studies are needed to better elucidate the precise role of the TNF rs1800629 polymorphism in cervical cancer.

Our findings are similar to those reported by Stanczuk et al. [15] and Govan et al. [16], who demonstrated no significant association between rs1800629 polymorphism and cervical cancer. Our findings of genotype distribution and HPV infection risk are supported by Deshpande et al. [14], who investigated the association of rs1800629 polymorphism with HPV 16 infection. However, their studies were based on a small sample size and might not have had sufficient statistical power to detect a difference. Although we have a large sample size, we were unable to confirm the possible genetic susceptibility of this SNP site.

Kirkpatrick et al. [22] demonstrated that cervical neoplasia was influenced by rs1800629 genotype. Since the cervical neoplasia tended to regress or progress spontaneously [23], it could affect the evaluation of risk of developing cervical cancer. Longer follow-up would have improved this detection rate.

The ethnic background in a population based genetic susceptibility study is also an important factor affecting the results. The genotype distribution of rs1800629 in our Chinese patients was similar to that reported in Indian [21], Korean [24] and Costa Rica Caucasian [25] populations, while our genotype distribution was slightly different from South Africa [15] and Zimbabwean [15] African, and USA [13], [23] and Mexico [14]. Caucasian populations. In a meta-analysis, Liu et al. [26] observed that the association between the rs1800629 AA homozygous genotype and cervical cancer was more significant in Asians. However, the studies groups cited by Liu et al. [26] were relatively small and under-powered.

Environmental exposures may also play an important role in these inconsistent results. Geographic variations in HPV type [27] may contribute to the different cervical cancer risks and alter the effect of rs1800629 polymorphism. However, our results did not support the relationship between HPV infection and rs1800629 polymorphism in the Chinese population.

One limitation of the present study was that it was a hospital-based, case control study. Patients were selected from a single institution and thus may not have been representative of cervical cancer patients in the general population. Control subjects were selected from the same hospital, also possibly introducing a selection bias. However, the distribution of rs1800629 genotype frequencies in our patients was similar to other reports [28] of the Chinese population, which suggested there was no selection bias.

TNF-α rs1800629 allele A tend to increase the expression of TNF-α [12], but evolution pressure seems to lower the frequency of high TNF-α producer, allele A, to reduce the mortality induced by high level of TNF-α. Considering our results, we postulate that in the immune compromised complex microbial vaginal environment, TNF-α doesn't play an important role in both HPV eliminating and HPV-related transformation. And in the progression of multiple factors induced cancer, the duality of TNF-α determine that it doesn't have decisive influence in cervical cancer.

### Conclusion

In our study, we found no association between TNF rs1800629 polymorphism and HPV infection, or cervical cancer with HPV infection.

## Supporting Information

Figure S1 **PCR-RFLP analysis and sequencing of TNF-alpha rs1800629 genotype.** (A) PCR-RFLP analysis results (electrophoresis on 3% Gel) (B) Sequencing results by ABI 3700.
(TIF)
