# Supplementary figures and images for: TNF-Alpha rs1800629 Polymorphism Is Not Associated with HPV Infection or Cervical Cancer in the Chinese Population
Source: PLoS One. 2012 Sep 13;7(9):e45246. doi: 10.1371/journal.pone.0045246 (PMC3441631; doi:10.1371/journal.pone.0045246)

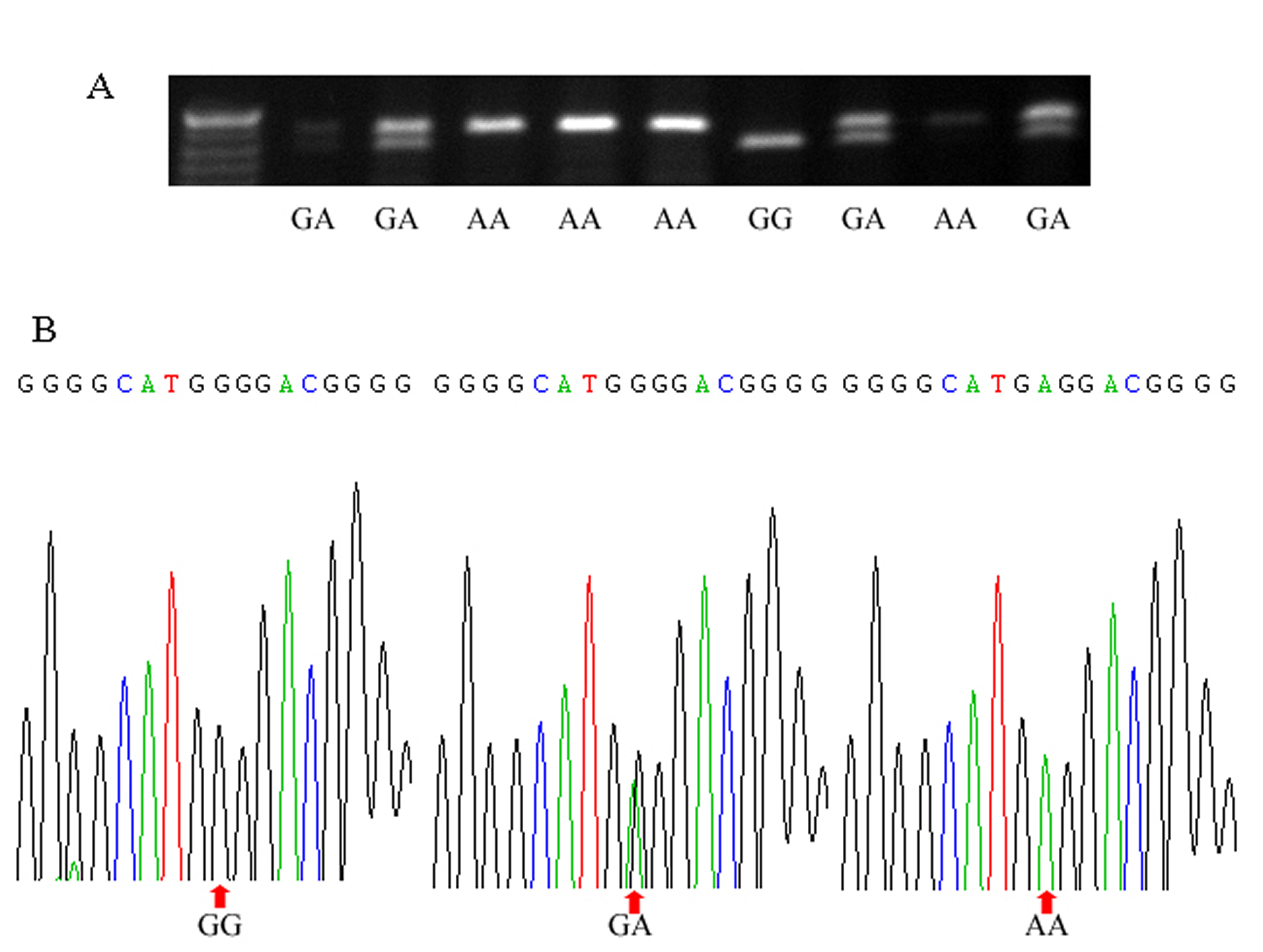

Supplement: Figure S1 — PCR-RFLP analysis and sequencing of TNF-alpha rs1800629 genotype. (A) PCR-RFLP analysis results (electrophoresis on 3% Gel) (B) Sequencing results by ABI 3700. (TIF) [file pone.0045246.s001.tif]
